# A Narrative Review of the Interplay Between Carbohydrate Intake and Diabetes Medications: Unexplored Connections and Clinical Implications

**DOI:** 10.3390/ijms26020624

**Published:** 2025-01-13

**Authors:** Mabitsela Hezekiel Mphasha, Rajesh Vagiri

**Affiliations:** 1 Department of Public Health, University of Limpopo, Mankweng 0727, South Africa; 2 Department of Pharmacy, University of Limpopo, Mankweng 0727, South Africa

**Keywords:** carbohydrate intake, diabetes management, insulin and metformin, glycemic index, continuous glucose monitoring, nutrient–drug interactions

## Abstract

This narrative review examines the dynamic interplay between carbohydrate intake and diabetes medications, highlighting their combined molecular and clinical effects on glycemic control. Carbohydrates, a primary energy source, significantly influence postprandial glucose regulation and necessitate careful coordination with pharmacological therapies, including insulin, metformin, glucagon-like peptide (GLP-1) receptor agonists, and sodium-glucose cotransporter-2 (SGLT2) inhibitors. Low-glycemic-index (GI) foods enhance insulin sensitivity, stabilize glycemic variability, and optimize medication efficacy, while high-GI foods exacerbate glycemic excursions and insulin resistance. Continuous glucose monitoring (CGM) offers real-time insights to tailor dietary and pharmacological interventions, improving glycemic outcomes and reducing complications. Despite advancements, gaps persist in understanding nutrient–drug interactions, particularly with emerging antidiabetic agents. This review underscores the need for integrating carbohydrate-focused dietary strategies with pharmacotherapy to enhance diabetes management. Future research should prioritize clinical trials leveraging CGM technology to explore how glycemic index, glycemic load, and carbohydrate quality interact with newer diabetes medications. Such studies can refine evidence-based recommendations, support individualized care plans, and improve long-term outcomes. Addressing systemic barriers, such as limited access to dietitians and CGM technology in underserved regions, is critical for equitable care. Expanding the roles of community health workers and training healthcare providers in basic nutrition counseling can bridge gaps, promoting sustainable and inclusive diabetes management strategies. These efforts are essential for advancing personalized, effective, and equitable care for individuals with diabetes.

## 1. Introduction

Diabetes mellitus, a chronic metabolic disorder marked by persistent hyperglycemia, represents an escalating global health challenge, currently affecting an estimated 537 million individuals worldwide [1]. Its prevalence is expected to rise due to urbanization, sedentary lifestyles, dietary transitions, genetic predisposition, and aging populations [2]. This growing burden places significant strain on healthcare systems, with complications such as cardiovascular disease, neuropathy, and renal failure contributing to increased morbidity and mortality [3]. Notably, populations in low- and middle-income countries, as well as marginalized groups in affluent nations, bear a disproportionate burden due to limited healthcare access, suboptimal diets, and low physical activity levels [4,5,6]. In South Africa, for instance, limited dietary knowledge among diabetic patients has been shown to significantly impact dietary practices and glycemic control, further exacerbating the challenges of managing the disease in resource-constrained settings [7].

Effective diabetes management hinges on the regulation of blood glucose levels, achieved through a combination of dietary modification and pharmacological interventions. Carbohydrates, as the primary energy source, are pivotal in this context, directly influencing glycemic control [8,9]. On a molecular level, the metabolism of carbohydrates into glucose, facilitated by glucose transporter 4 (GLUT4) under insulin regulation, underscores the necessity of balancing carbohydrate intake to mitigate glycemic excursions and prevent complications [10].

Dietary strategies emphasizing fiber-rich foods, whole grains, and low-glycemic-index options play a critical role in modulating postprandial glucose responses, thereby enhancing insulin sensitivity [8,11,12,13]. The delayed absorption of glucose through the intestinal tract and the enhanced signaling of insulin receptors exemplify the molecular mechanisms underpinning these effects [14]. Careful monitoring of carbohydrate intake, alongside an appropriate balance of proteins and fats, is essential for minimizing glycemic variability and optimizing metabolic outcomes [15,16].

Pharmacological management complements dietary efforts through a diverse array of medications tailored to individual needs. Metformin, the first-line treatment for type 2 diabetes, suppresses hepatic gluconeogenesis via AMP-activated protein kinase (AMPK) activation, reducing blood glucose concentrations [17]. Sulfonylureas and meglitinides promote insulin secretion by modulating ATP-sensitive potassium channels in pancreatic beta cells, while sodium-glucose cotransporter-2 (SGLT2) inhibitors facilitate glucose excretion through the kidneys. Additionally, glucagon-like peptide-1 (GLP-1) receptor agonists enhance insulin release and inhibit glucagon secretion through cAMP signaling pathways [18,19,20,21,22]. Insulin therapy remains indispensable for type 1 diabetes and advanced type 2 diabetes, facilitating glucose uptake through GLUT4 translocation to the cell membrane [23].

A critical yet underexplored aspect of diabetes management is the synchronization of carbohydrate intake with medication regimens. While it is well established that carbohydrate timing, quality, and quantity influence the efficacy of pharmacological treatments, real-world evidence integrating structured dietary interventions with diabetes medications remains scarce [24,25]. For instance, carbohydrate counting and accurate meal timing are essential in insulin therapy to align insulin action with glucose availability [15]. Similarly, metformin is often administered with meals to mitigate gastrointestinal side effects, exemplifying the importance of integrating dietary practices with medication use [26]. Despite these recognized interactions, Shaughnessy [27] highlights in the updated American Diabetes Association (ADA) and European Association for the Study of Diabetes (EASD) guidelines that no standardized clinical trials have systematically evaluated the direct impact of specific carbohydrate compositions on the effectiveness of diabetes medications in routine patient management

The glycemic index (GI) and glycemic load (GL) provide essential tools for understanding the impact of carbohydrates on postprandial glucose levels [23,28]. Measures such as the oral glucose tolerance test (OGTT) and area under the curve (AUC) offer valuable insights into glucose metabolism [29]. However, the application of these dietary and pharmacological strategies in research remains underexplored. Continuous glucose monitoring (CGM) technology has revolutionized diabetes care by providing real-time glucose data, yet few studies have systematically utilized CGM to assess the direct relationship between carbohydrate intake and medication efficacy. Recent discussions at the 60th EASD Annual Meeting [30] emphasized the need for integrating CGM-based research to better understand these interactions and optimize diabetes treatment strategies. A comprehensive evaluation of nutrient–drug interactions using CGM data could enhance personalized diabetes management strategies and optimize glycemic control.

Despite significant advances, substantial gaps remain in understanding the interactions between carbohydrate quality, GI/GL, and emerging diabetes medications. The current evidence highlights the importance of carbohydrate management in glycemic control, but data on the dynamic interplay between dietary factors and newer pharmacological therapies are limited. This narrative review addresses these gaps by critically examining molecular and clinical evidence on the interaction between carbohydrate intake and diabetes medications. By consolidating the current knowledge and identifying research priorities, this review aims to strengthen evidence-based guidelines, equipping healthcare providers with the insights needed to offer tailored dietary recommendations. This approach will empower patients to achieve optimal glycemic control and long-term health outcomes through a more integrated and personalized diabetes management strategy.

## 2. Method

A narrative review was conducted to explore the complex interactions between carbohydrate intake and diabetes medications at both clinical and molecular levels. Narrative reviews serve as a valuable tool for synthesizing diverse research methodologies, including qualitative, quantitative, and mixed-method studies, into a cohesive analysis that offers a holistic understanding of intricate topics. A structured approach was employed to ensure methodological rigor and comprehensiveness.

### 2.1. Literature Search

Comprehensive literature searches were performed using PubMed, Scopus, and Web of Science. Additionally, authoritative guidelines from professional organizations such as the American Diabetes Association (ADA), International Diabetes Federation (IDF), Society for Endocrinology, Metabolism, and Diabetes of South Africa (SEMDSA), European Association for the Study of Diabetes (EASD), the Academy of Nutrition and Dietetics (AND), and the World Health Organization were included. Keywords included “carbohydrate quality”, “glycemic index”, “glycemic load”, “diabetes medications”, “nutrient-drug interactions”, and “continuous glucose monitoring.” Inclusion criteria encompassed clinical trials, systematic reviews, meta-analyses, and molecular studies exploring the interaction between carbohydrates and diabetes medications. Exclusion criteria included studies that focused solely on the efficacy of diabetes medications without considering dietary influences or those that examined carbohydrate intake without assessing its interaction with pharmacotherapy. Additionally, studies that investigated general nutrition or lifestyle interventions without specific reference to carbohydrate metabolism and its impact on diabetes medications were excluded. Research that lacked clear glycemic outcome measures, such as CGM, GI, or GL, was also omitted to maintain the review’s focus on integrative strategies for diabetes management.

### 2.2. Scope

To ensure comprehensiveness, no restrictions were placed on the publication period. This allowed the integration of foundational research alongside recent advances in diabetes care. This inclusive methodology facilitated the examination of key molecular mechanisms, clinical outcomes, and emerging therapeutic strategies. This review particularly aimed to address gaps in current knowledge, focusing on the impact of carbohydrate quality on the pharmacological actions of newer diabetes medications such as SGLT2 inhibitors and GLP-1 receptor agonists.

### 2.3. Research Questions

The review was guided by the following research questions:

- How important are carbohydrates in diabetes management?
- What medications are commonly used to control diabetes?
- How does carbohydrate intake influence the effectiveness and safety of specific diabetes medications?

## 3. Glycemic Index, Glycemic Load, and Carbohydrate Quality

The quality and type of carbohydrates consumed play a critical role in regulating blood glucose levels and modulating the effectiveness of diabetes medications. At the molecular level, factors such as GI, fiber content, and nutrient composition influence the rate of glucose absorption, insulin secretion, and insulin signaling pathways, directly impacting glycemic control and the pharmacodynamics of antidiabetic therapies [31].

Carbohydrates with a low GI produce a slower and more sustained release of glucose into the bloodstream. This is largely due to the structural properties of fiber and the biochemical interactions that inhibit rapid enzymatic digestion. Whole grains, such as brown rice, quinoa, oats, and whole wheat bread, exemplify low-GI foods due to their high fiber content and intact grain structures. These structural components physically impede digestive enzyme access, slowing carbohydrate hydrolysis and glucose absorption [32]. Additionally, fiber fermentation by gut microbiota produces short-chain fatty acids (SCFAs), such as butyrate, which enhance insulin sensitivity by modulating inflammation and activating signaling pathways like AMPK [32,33].

Legumes, including beans, lentils, and chickpeas, are particularly effective in glycemic control due to their high fiber and protein content, which further delay glucose absorption. At the cellular level, these foods enhance GLUT4 translocation to the plasma membrane in response to insulin, facilitating stable glucose uptake and reducing glycemic variability [34,35]. In contrast, high-GI foods such as refined grains and sugary beverages induce rapid spikes in blood glucose levels, placing strain on the insulin signaling pathway, increasing oxidative stress, and contributing to beta-cell dysfunction and insulin resistance over time [28,36].

The nutrient density of legumes, low-GI fruits, and non-starchy vegetables not only stabilizes blood glucose levels but also improves metabolic health by mitigating hyperglycemia-induced inflammation and oxidative stress. These foods play a vital role in diabetes management by promoting sustained glycemic control and reducing the risk of long-term complications [32,33].

## 4. Oral Glucose Tolerance, Test Area Under the Curve, and Carbohydrate Intake

The OGTT is a vital diagnostic tool for evaluating glucose metabolism and glycemic control by measuring the body’s ability to process a standardized glucose load [34]. It provides insights into key physiological processes, including insulin secretion, hepatic glucose output, and peripheral glucose uptake, highlighting the interplay between beta-cell function and insulin sensitivity. OGTT also identifies conditions such as impaired fasting glucose (IFG), impaired glucose tolerance (IGT), and diabetes mellitus, making it valuable for monitoring therapeutic and lifestyle interventions [35].

AUC, derived from OGTT or continuous glucose monitoring data, reflects total glucose exposure over time. A high AUC often indicates prolonged hyperglycemia and oxidative stress, which can lead to cardiovascular and microvascular complications [36]. AUC is particularly useful for evaluating interventions targeting glucose metabolism and managing postprandial hyperglycemia, which is associated with advanced glycation end-product (AGE) formation and tissue damage [37].

Integrating OGTT and AUC with carbohydrate management strategies enhances glycemic control by enabling personalized dietary interventions. These tools allow for precise evaluation of glucose responses to specific carbohydrate sources and help mitigate risks associated with fasting and postprandial hyperglycemia, reducing complications like neuropathy and cardiovascular disease [38].

Despite their utility, OGTT and AUC have limitations, including resource intensity, interindividual variability in glucose tolerance, and the complexity of translating findings into practical dietary guidelines [34,39]. Future research should aim to develop non-invasive alternatives to OGTT, standardize AUC methodologies, and investigate the molecular effects of carbohydrate quality. Incorporating technologies like continuous glucose monitoring could further enhance their clinical applicability.

Together, OGTT, AUC, and carbohydrate management strategies offer a robust framework for personalized glycemic control, improving patient outcomes and advancing molecular understanding of glucose metabolism. However, addressing their limitations through innovation and research is crucial for maximizing their potential.

## 5. Oral Antidiabetic Medications and Molecular Mechanisms

Oral antidiabetic medications target distinct molecular pathways to regulate glucose homeostasis, often complementing the effects of carbohydrate intake. The interplay between these medications and dietary carbohydrates is crucial for optimizing glycemic control and minimizing adverse effects.

**Insulin secretagogues (sulfonylureas and meglitinides):** These medications enhance insulin secretion by binding to ATP-sensitive potassium (KATP) channels on pancreatic beta cells, triggering membrane depolarization and calcium influx, which stimulates insulin release [40]. The ingestion of carbohydrates raises blood glucose, augmenting this insulinotropic effect. However, precise carbohydrate monitoring is essential to avoid hypoglycemia, as excess insulin release can occur when carbohydrate intake is insufficient to match insulin secretion [41,42].

**Insulin sensitizers (thiazolidinediones and metformin):** Thiazolidinediones activate peroxisome proliferator-activated receptor gamma (PPARγ), enhancing glucose uptake in adipose tissue and skeletal muscle while promoting lipid metabolism [43]. Metformin, a cornerstone in type 2 diabetes management, activates AMPK, suppressing hepatic gluconeogenesis and increasing glucose uptake in peripheral tissues [44]. These pathways benefit from stable blood glucose levels facilitated by low-GI diets, which enhance the efficacy of insulin sensitizers by reducing glucose flux variability [45,46].

**Alpha-glucosidase inhibitors:** These medications inhibit alpha-glucosidase enzymes in the small intestine, delaying the breakdown of polysaccharides into monosaccharides and attenuating postprandial glucose spikes. The timing of medication administration with meals is critical to synchronizing enzymatic inhibition with carbohydrate digestion, optimizing postprandial glycemic control [47].

**Sodium-glucose cotransporter-2** (**SGLT2) inhibitors:** SGLT2 inhibitors reduce renal glucose reabsorption, while GLP-1 receptor agonists enhance insulin secretion and delay gastric emptying. These mechanisms interact with dietary carbohydrates, impacting postprandial glucose levels and overall glycemic variability [18]. However, limited studies explicitly examine these interactions using CGM or carbohydrate counting as primary endpoints.

Beyond these general interactions, specific antidiabetic medications exhibit unique responses to carbohydrate intake. For instance, patients using orlistat may experience worsened digestive side effects, such as bloating and diarrhea, when consuming high-carbohydrate meals due to its impact on fat absorption [48]. GLP-1 receptor agonists, while effective in regulating glycemia, may increase the risk of nausea and vomiting when paired with excessive carbohydrate intake [22,49]. Similarly, SGLT2 inhibitors may have their glucose-lowering efficacy diminished by increased carbohydrate consumption, which can alter renal glucose excretion efficiency. These clinically significant interactions further highlight the necessity for individualized dietary recommendations.

Effective carbohydrate monitoring is critical for optimizing the use of oral antidiabetic medications. Quantifying carbohydrate intake enables patients and healthcare providers to adjust medication dosages appropriately, minimizing glycemic excursions and the risk of hypoglycemia, particularly with insulin secretagogues. Regular blood glucose monitoring helps identify patterns in glycemic responses, allowing for personalized adjustments to dietary and pharmacological regimens to achieve precise glycemic control [9,50]. However, the scarcity of formal studies investigating specific carbohydrate compositions and their interaction with various diabetes medications presents a critical gap in the current research. Addressing this gap is vital for refining dietary strategies to maximize therapeutic benefits while minimizing adverse outcomes.

## 6. Insulin Treatment

Insulin therapy is intrinsically linked to carbohydrate intake in diabetes management, with both physiological and molecular interactions playing a pivotal role in maintaining glycemic control [12]. Insulin, a peptide hormone produced by pancreatic beta cells, is crucial for facilitating glucose uptake into cells by promoting the translocation of glucose transporter 4 (GLUT4) vesicles to the plasma membrane. This molecular process is vital for ensuring blood glucose homeostasis, particularly in response to carbohydrate consumption. The quantity and quality of carbohydrates ingested directly influence insulin requirements, making precise insulin dosing a cornerstone of effective diabetes management [51,52].

### 6.1. Carbohydrate Counting and Insulin-to-Carbohydrate Ratio

One of the key strategies for aligning insulin dosing with carbohydrate intake is carbohydrate counting. This method involves estimating the number of grams of carbohydrates in a meal to determine the appropriate insulin dose required to manage postprandial glucose fluctuations. The insulin-to-carbohydrate ratio (ICR) is an essential tool in this process, where a ratio such as 1:10 indicates that one unit of insulin is needed for every 10 g of carbohydrates consumed [53]. By matching insulin doses to the carbohydrate load, this approach optimizes insulin signaling pathways, helping to avoid both hyperglycemia and hypoglycemia. This molecular precision ensures that glucose uptake occurs in a balanced manner, enhancing the overall effectiveness of diabetes management.

### 6.2. Insulin Timing and Glycemic Control

The timing of insulin administration is a critical aspect of diabetes management, particularly in relation to the absorption rates of carbohydrates. Rapid-acting insulin analogs such as lispro and aspart are typically administered shortly before meals to address the swift postprandial glucose rise. These insulins act within 15 min, aligning with the rapid glucose absorption phase following carbohydrate ingestion [54]. In contrast, short-acting insulin is administered approximately 30 min before meals, due to its slower onset of action, providing a more gradual effect on glucose regulation [55]. Intermediate-acting insulins like NPH are used for basal glucose control and are not directly influenced by carbohydrate intake, although they support overall glycemic stability [56].

At the molecular level, insulin binds to its receptor, activating the PI3K-AKT signaling cascade, which facilitates GLUT4 vesicle fusion with the cell membrane, thereby enabling glucose uptake. The rate of this glucose uptake is influenced by the GI of the carbohydrate consumed. Low-GI carbohydrates, which release glucose more gradually, provide a sustained substrate for insulin action, reducing glycemic variability and enhancing insulin efficacy. Conversely, high-GI foods, which cause rapid glucose spikes, require precise insulin timing to mitigate hyperglycemia and prevent insulin resistance [53,57].

### 6.3. Molecular Dynamics of Carbohydrate Absorption and Insulin Action

Carbohydrate quality, particularly in terms of glycemic index and fiber content, plays a key role in modulating insulin action at the molecular level. Low-GI carbohydrates such as whole grains, legumes, and non-starchy vegetables release glucose slowly, thereby reducing insulin spikes and allowing for a more gradual and sustained activation of GLUT4, which promotes efficient glucose uptake. This steady release helps stabilize postprandial glucose levels, enhancing the synergistic action between insulin and glucose transport mechanisms.

In contrast, high-GI carbohydrates, such as refined grains and sugary foods, cause rapid glucose absorption, triggering quick insulin secretion [58]. These fluctuations strain the insulin signaling pathway and increase the risk of insulin resistance and beta-cell exhaustion over time. Therefore, managing the type and quantity of carbohydrates consumed is essential to achieving stable glycemic control and optimizing the pharmacodynamics of insulin therapy.

## 7. Diet and Single-Medication Strategies in Diabetes Management

Dietary intervention, with a primary focus on carbohydrate intake, is the cornerstone of diabetes management and serves as the most impactful initial step in controlling blood glucose levels. Carbohydrate-focused strategies, such as incorporating low-GI foods, increasing dietary fiber, and balancing macronutrients, play a pivotal role in regulating postprandial glucose excursions and improving insulin sensitivity [12,13]. These approaches modulate critical molecular pathways, including AMPK activation and GLUT4 translocation, to address metabolic dysfunctions effectively [32]. However, as diabetes progresses or adherence diminishes, diet alone may not suffice, necessitating the addition of pharmacological therapies to achieve optimal glycemic control [59].

Studies evaluating the integration of carbohydrate-focused dietary modifications with a single medication highlight the synergistic effects of these combined approaches [60,61,62]. Metformin, when paired with a low-GI diet, enhances insulin sensitivity and reduces glycemic variability by stabilizing postprandial glucose levels [63]. GLP-1 receptor agonists, when combined with high-fiber diets, amplify satiety signals and improve glucose regulation by slowing carbohydrate absorption [64]. Similarly, SGLT2 inhibitors, used alongside carefully managed carbohydrate intake, promote glycemic control by facilitating renal glucose excretion [65]. For patients on insulin therapy, precise carbohydrate management, including carbohydrate counting and prioritizing low-GI foods, ensures accurate dosing and minimizes the risks of hypoglycemia and hyperglycemia [52].

These underscore the central role of carbohydrate intake in diabetes management and the importance of personalized treatment strategies that integrate dietary and pharmacological interventions. The total diet approach, as recommended by the Academy of Nutrition and Dietetics [16], underscores the need to consider overall dietary patterns, rather than focusing solely on individual nutrients, to enhance glycemic control and patient adherence. In South Africa, the Society for Endocrinology, Metabolism and Diabetes of South Africa (SEMDSA) Guidelines [66] provide evidence-based recommendations for integrating dietary strategies with pharmacological interventions to improve glycemic control. Healthcare providers, particularly dietitians, are essential in designing tailored plans that align carbohydrate intake with individual metabolic profiles and treatment goals. Future research should emphasize the long-term effects of carbohydrate-focused dietary patterns combined with emerging diabetes medications, with a focus on the molecular mechanisms that underpin these synergistic effects. This will provide a foundation for robust, evidence-based guidelines to optimize diabetes care.

## 8. Carbohydrate Monitoring and Personalized Treatment

Monitoring carbohydrate intake is crucial for optimizing the coordination between diet and insulin therapy. Monitoring carbohydrate consumption, blood glucose levels, and insulin doses provides valuable data for adjusting insulin regimens and improving glycemic control [59,67]. Prevention-focused strategies outlined by the Academy of Nutrition and Dietetics Evidence Analysis Library [68] emphasize early intervention with carbohydrate-focused dietary changes to mitigate the progression of diabetes. Patients can use tools such as food journals, digital glucose meters, and mobile apps to record their meals and monitor their blood glucose responses. This information helps to refine insulin dosing strategies, ensuring that insulin-to-carbohydrate ratios are optimized for individual needs [36].

Modern technologies like CGM provide real-time data on glycemic fluctuations, offering insights into the interplay between diet and medication. For example, studies utilizing CGM have shown that low-GI diets reduce glycemic variability in individuals using SGLT2 inhibitors [69,70]. CGM devices integrate information about carbohydrate intake, insulin administration, and blood glucose levels, allowing patients to identify patterns and make informed adjustments to both their diet and medication [71]. These devices help detect delayed hyperglycemia or nocturnal hypoglycemia, further improving glycemic stability and reducing the risks of complications [72,73].

By leveraging these data, healthcare providers can create personalized treatment plans that optimize insulin-to-carbohydrate ratios and adjust medication schedules based on individual glycemic responses. The integration of continuous monitoring with precise insulin management ensures better long-term outcomes, enhanced quality of life, and a reduced risk of diabetes-related complications [69]. This molecular and clinical synergy between carbohydrate intake and insulin therapy emphasizes the importance of a tailored, data-driven approach to diabetes management.

Table 1 categorizes carbohydrate sources based on their GI and their effects on blood sugar levels. Low-GI foods like whole grains, non-starchy vegetables, and legumes are recommended for diabetes patients as they provide a slower, more stable glucose release. High-GI foods like refined carbohydrates can cause quick spikes in blood glucose levels, complicating blood sugar control.

## 9. Individualized Strategy for Carbohydrate Intake and Medication Interactions

Achieving optimal blood sugar control in diabetes requires an individualized approach that accounts for both carbohydrate intake and medication interactions. At the molecular level, the body’s response to carbohydrates involves intricate interactions between insulin signaling pathways, glucose transporters, and cellular metabolism. These molecular processes are influenced by various factors, including age, weight, physical activity, medication regimen, and personal blood sugar goals. The variations in response to carbohydrates and insulin can also stem from differences in insulin sensitivity, genetic predispositions, and environmental exposures [74].

### 9.1. Molecular Basis for Individualized Approaches

The molecular variability in how individuals process carbohydrates is crucial for developing tailored strategies. Insulin sensitivity and glucose metabolism differ widely across individuals, influencing how efficiently glucose is absorbed and utilized. These individual differences can be attributed to genetic factors such as polymorphisms in the insulin receptor or glucose transporter genes, as well as environmental influences like diet, exercise, and stress levels [44]. For example, individuals with higher genetic predispositions to insulin resistance may need more precise carbohydrate management to avoid exacerbating their condition. Understanding these molecular nuances enables healthcare providers to personalize dietary recommendations and medication regimens to better match an individual’s unique metabolic profile.

### 9.2. Role of Dietitians in Individualized Carbohydrate Management

A trained dietitian plays a pivotal role in helping individuals manage carbohydrate intake based on their unique molecular responses. Dietitians guide patients through carbohydrate counting and education on how different carbohydrates impact insulin secretion and glucose uptake at the cellular level [75]. By emphasizing the molecular dynamics between carbohydrate digestion, absorption, and insulin sensitivity, dietitians can design customized meal plans that reduce glycemic variability. For instance, incorporating low-GI foods such as whole grains and legumes into a patient’s diet promotes a slower glucose release, which stabilizes postprandial blood glucose levels and enhances insulin signaling pathways, improving overall insulin sensitivity.

At the molecular level, foods with low GI promote more gradual glucose absorption, which influences the activation of insulin receptor signaling pathways and reduces the strain on insulin-producing beta cells [76]. Conversely, high-GI foods trigger rapid glucose spikes, overstimulating insulin secretion and leading to insulin resistance over time [77]. By carefully selecting foods that prioritize low GI and high fiber content, dietitians help manage blood sugar fluctuations and reduce inflammation at the cellular level, mitigating the risk of postprandial hyperglycemia [78].

Despite the critical role dietitians play in promoting health and managing chronic diseases, their utilization in underserved regions is hindered by workforce shortages, geographic disparities, and limited awareness of their benefits among communities and healthcare providers. Diabetes self-management education programs are critical for empowering patients to take an active role in their care by equipping them with the knowledge and skills to manage carbohydrate intake and medication effectively [79]. Addressing these barriers requires targeted interventions, including equipping primary care providers and community health workers (CHWs) with basic nutrition training to deliver practical and culturally relevant dietary advice [78]. Telehealth platforms can bridge geographical gaps, enabling remote dietetics consultations and follow-ups. Additionally, integrating dietitian services into diabetes programs and school-based nutrition efforts can broaden their reach. Raising community awareness through workshops and collaborations with local leaders, alongside deploying mobile units for on-site services, further ensures access to nutrition care in remote areas. These strategies collectively aim to enhance dietitian utilization and improve health outcomes in underserved communities as advocated by the World health Organization (WHO) [80].

### 9.3. Glycemic Load and Insulin Sensitivity

While the glycemic index measures the quality of carbohydrates, GL provides a more comprehensive assessment by incorporating both quality and quantity. High-GL foods can induce rapid glucose spikes that overwhelm the insulin response and diminish the effectiveness of medications like GLP-1 receptor agonists or insulin secretagogues [81]. In contrast, low-GL foods promote a gradual insulin release, enhancing glucose homeostasis and reducing oxidative stress, key factors in mitigating insulin resistance [82]. Tailored dietary interventions that focus on glycemic load can therefore optimize medication performance while minimizing adverse effects. Recent advancements presented at the 60th EASD Annual Meeting [30] emphasize the role of CGM in understanding and optimizing the interaction between glycemic load and medication efficacy in diabetes management.

### 9.4. Continuous Glucose Monitoring for Real-Time Adjustments

Incorporating CGM into individualized treatment plans offers significant benefits for tracking the effects of carbohydrate intake on blood glucose levels in real-time [83]. CGM devices provide dynamic, real-time insights into how specific foods and medications influence glucose levels throughout the day [84]. This technology helps reveal how carbohydrate timing, quality, and quantity interact with insulin administration to influence blood glucose fluctuations [85]. By continuously monitoring glucose trends, patients can adjust their diet and medication in response to changing blood sugar patterns, ensuring more precise management of their diabetes [33].

CGM technology enables a more nuanced understanding of the molecular interactions between dietary intake and medication, allowing patients to identify delayed hyperglycemia or nocturnal hypoglycemia, which may not be evident through traditional finger-stick testing [86]. A study by Franz et al. reinforces the value of CGM in integrating dietary strategies, such as low-GI diets, with pharmacological interventions to optimize glycemic control and patient outcomes [33]. Real-time data enables healthcare providers to adjust insulin-to-carbohydrate ratios and refine meal planning strategies, ensuring optimal glucose control and reducing the risk of diabetes-related complications [62].

Despite advancements in understanding carbohydrate-medication interactions, significant research gaps remain. Few studies have systematically examined how specific carbohydrate compositions influence the pharmacodynamics of newer antidiabetic agents like GLP-1 receptor agonists and SGLT2 inhibitors. Future research should prioritize clinical trials that leverage CGM technology to explore these interactions, providing robust evidence for refining dietary guidelines and optimizing medication efficacy. Filling these gaps will empower healthcare providers to deliver more personalized, evidence-based care that aligns dietary and pharmacological strategies for improved diabetes management.

Table 2 underscores the critical role of carbohydrate quality, glycemic index categorization, and precise medication adjustments in effective diabetes management. Emphasizing low-GI foods helps stabilize blood glucose and reduce post-meal spikes. Consistent monitoring, paired with a personalized approach to diet and pharmacotherapy, ensures optimal glycemic control and improved patient outcomes.

Table 3 provides a concise summary of the synergistic effects observed when combining a low-GI diet with various diabetes medications.

## 10. Conclusions

This review highlights the intricate yet underexplored interplay between carbohydrate intake and diabetes medications, emphasizing the need for a more integrated approach to diabetes management. While the existing evidence underscores the importance of carbohydrate quality, glycemic index, and glycemic load in glycemic control, real-world data on how specific carbohydrate compositions influence medication efficacy remain scarce. The current research lacks standardized clinical trials that assess the direct impact of dietary carbohydrates on the pharmacodynamics of diabetes medications in routine patient care.

To advance personalized diabetes management, future studies should leverage CGM and carbohydrate counting as primary outcomes to provide real-time insights into nutrient–drug interactions. Additionally, well-designed dietary intervention trials are needed to measure glucose fluctuations in response to different carbohydrate sources in patients using various diabetes medications. Such studies could help refine dietary guidelines, optimize medication efficacy, and reduce glycemic variability, ultimately improving patient outcomes. Emerging diagnostic tools, such as the 1 h post-load plasma glucose test discussed by Bergman et al., represent a promising avenue for early detection and monitoring of glycemic patterns. These advancements could complement future research efforts in integrating diagnostics with personalized dietary and pharmacological strategies [87].

By addressing these research gaps, future investigations can strengthen evidence-based recommendations for clinicians, ensuring that dietary and pharmacological strategies are aligned to achieve optimal glycemic control [88]. A more structured, data-driven approach will not only enhance diabetes care but also empower patients with tailored dietary strategies that complement their prescribed treatments. As Shaughnessy [27] highlights, broader health outcomes—encompassing cardiovascular health, lifestyle modifications, and patient education—must also be integrated into comprehensive care plans to ensure long-term well-being and quality of life.

## 11. Limitations

This review acknowledges several limitations in the current research on carbohydrate and medication interactions in diabetes management. One significant challenge is the lack of standardization across studies, which hinders the ability to compare findings and draw definitive conclusions. Moreover, there is a knowledge gap regarding the interactions between newer classes of diabetes medications, such as SGLT-2 inhibitors and GLP-1 receptor agonists, with specific types of carbohydrates. Furthermore, the quality of carbohydrates—including factors like food processing and individual variations in digestion rates—adds complexity to understanding how carbohydrates influence glucose metabolism at a molecular level.

Confounding variables, such as dietary patterns, physical activity, and other health conditions, can also affect the outcomes of diabetes treatments and contribute to variability in individual responses. Additionally, many studies focus on short- to medium-term effects, leaving a gap in our understanding of long-term outcomes related to carbohydrate intake and medication interactions. The reliance on self-reported data introduces potential inaccuracies and biases, particularly when patients may struggle with accurate food tracking or glucose monitoring. Finally, cultural and regional differences in diet and lifestyle can affect the generalizability of findings, limiting the broader applicability of current research. Addressing these limitations through more rigorous, standardized studies and more comprehensive data collection will be crucial for advancing the understanding of carbohydrate and medication interactions in diabetes management.

## Figures and Tables

**Table 1:** Common carbohydrate sources and their glycemic index (GI) impact.

Carbohydrate Source	GI Category	Molecular Effect on Blood Sugar	Examples	Additional Benefits
Whole grains	Low	Gradual rise in blood sugar due to delayed glucose absorption and improved insulin sensitivity via SCFAs.	Brown rice, whole wheat bread, oats	Rich in micronutrients and prebiotics, supporting gut health
Non-starchy vegetables	Low	Minimal glycemic impact due to high fiber content reducing carbohydrate digestion.	Spinach, broccoli, cauliflower	High in antioxidants and anti-inflammatory compounds
Legumes	Low	Slow glucose release mediated by fiber and protein content, reducing postprandial glucose variability.	Beans, lentils, chickpeas	Supports gut microbiome and cardiovascular health
Refined carbohydrates	High	Rapid glucose absorption overwhelms insulin pathways, leading to spikes in blood sugar.	White bread, pastries, sugary snacks	Often lacking essential nutrients and contributing to metabolic syndrome
Fruits (varies)	Mixed	Fiber in low-GI fruits moderates glucose absorption; high-GI tropical fruits cause rapid glucose spikes.	Apples, berries (low); mango, pineapple (high)	High in vitamins and phytonutrients, but portion control is key

**Table 2:** Summary of key findings.

Aspect	Key Findings	Emerging Perspectives
Carbohydrate quality	Incorporating whole grains, fruits, and legumes with a low GI enhances insulin sensitivity and maintains stable blood glucose levels by promoting slow, controlled glucose release.	Emerging research suggests that polyphenols in low-GI foods may further enhance insulin sensitivity.
Medication interaction	Insulin and oral antidiabetic agents (e.g., metformin, sulfonylureas) interact dynamically with carbohydrate intake, necessitating precise dose adjustments for optimal glucose regulation.	The effectiveness of medication can be influenced by meal composition beyond carbohydrate content, such as protein and fat intake.
High vs. low GI foods	Low-GI foods support gradual glucose release, enhancing insulin efficacy and improving postprandial glucose control, while high-GI foods cause rapid glucose spikes and increased glycemic variability.	Combining low-GI foods with resistant starches can further stabilize blood sugar responses.
Monitoring	Regular tracking of carbohydrate intake and blood glucose levels enables timely adjustments to medication dosages, minimizing the risks of hypo- and hyperglycemia.	Smart technology, such as AI-driven dietary recommendations, is enhancing personalized glucose management.
Individualized approach	Tailored carbohydrate and medication plans, designed to align with individual patient lifestyles and preferences, improve overall diabetes management and clinical outcomes.	Personalized microbiome analysis could refine dietary recommendations for improved glucose control.

**Table 3:** Key insights on low-GI diet and medication integration in diabetes management.

Intervention	Key Insights	Additional Considerations
Low-GI diet alone	Reduces glycemic variability and provides modest improvements in HbA1c.	Works best when combined with lifestyle modifications like physical activity.
Low-GI diet + Metformin	Enhances glycemic stability and HbA1c outcomes more effectively than diet alone.	Patients with gastrointestinal sensitivity may need alternative dietary approaches.
Low-GI diet + GLP-1 agonists	Offers the greatest HbA1c improvement and significantly reduces glycemic variability.	Fiber intake should be optimized to prevent gastrointestinal discomfort.
Low-GI diet + SGLT2 inhibitors	Combines the effects of renal glucose excretion with dietary impact for robust glycemic control.	Hydration status and electrolyte balance must be monitored.
